# Galactose induces formation of cell wall stubs and cell death in Arabidopsis roots

**DOI:** 10.1007/s00425-022-03919-x

**Published:** 2022-07-03

**Authors:** Margit Höftberger, Martina Althammer, Ilse Foissner, Raimund Tenhaken

**Affiliations:** Department of Environment & Biodiversity, Plant Physiology, All Paris-Lodron University Salzburg, Hellbrunnerstr. 34, 5020 Salzburg, Austria

**Keywords:** Binuclear cells, Ectopic lignification, Galactokinase, Galactose toxicity, Incomplete cell division, Root morphology

## Abstract

**Main conclusion:**

Arabidopsis seedlings growing on low concentration of galactose stop regular root growth. Incomplete cell division with cell wall stubs, binuclear and giant cells and lignified root tips are observed.

**Abstract:**

Galactose is a sugar abundant in root cell walls of Arabidopsis. Nevertheless, we found that the germination of Arabidopsis seedlings on galactose containing media causes a strong modification of the root development, as shown by analysing the root with microscopy methods ranging from the bright field over confocal to transmission electron microscopy. At concentrations of about 1 mM, the growth of the primary root stops after a few days though stem cell markers like *WOX5* are still expressed. The root tip swells and forms a slightly opaque, partially lignified structure in parts of the cortex and the central cylinder. The formation of the cell plate after mitosis is impaired, often leading to cell wall stubs and binuclear cells. Some cells in the cortex and the central cylinder degenerate, while some rhizodermal and cortical cells increase massively in size. The galactose toxicity phenotype in Arabidopsis depends on the activity of galactokinase and is completely diminished in galactokinase knock-out lines. From the comparison of the galactose toxicity phenotype with those of cytokinesis mutants and plants treated with appropriate inhibitors we speculate that the toxicity syndrome of galactose is caused by interference with intracellular vesicle transport or cell wall biogenesis.

**Supplementary Information:**

The online version contains supplementary material available at 10.1007/s00425-022-03919-x.

## Introduction

Galactose is an abundant sugar in nature. Galactose is found in several types of plant cell wall polymers but is also a component of the seed storage oligomers raffinose, stachyose and higher-order derivatives thereof (Gangl and Tenhaken 2016). During germination, raffinose and stachyose are metabolised to sucrose by galactosidases liberating millimolar amounts of galactose. During the development of seedlings, the cell wall is remodelled, which is associated with the release of galactose from cell wall polymers. The plant converts the liberated galactose to the corresponding nucleotide sugar with the enzymes galactokinase (Egert et al. 2012) and UDP-sugar pyrophosporylase (Kotake et al. 2004). This allows the recycling of liberated galactose for the biosynthesis of new cell wall material as well as for glycoproteins like the arabinogalactan-proteins.

Given the ubiquitous occurrence of galactose, it comes as a surprise, that galactose is toxic to plants as well as to fungi and animals. It has been known for over 100 years that galactose represses the growth of vetch (*Vicia villosa*) and other higher plants and that this effect is ameliorated in the presence of other sugars (Knudson 1915; compare Wang et al. 2015). Experiments with barley roots transferred to 10–20 mM galactose medium have shown increased uptake of assimilates for a few hours after transfer, followed by an inhibition of growth (Thorpe et al. 1999). This pattern was also found for other monocots, but not for eudicots. A decline in the elongation rate of Arabidopsis roots after transfer to 20 mM galactose was observed by Thorpe et al. (1999), without a transient increase of assimilates. The inhibition of root growth in galactose medium in maize was associated with cell wall tightening and an increase in turgor pressure (Pritchard et al. 2004). The inhibitory effect of galactose on hormone mediated extension rates of coleoptiles or hypocotyls from various plants was investigated by Yamamoto et al. (1988) and Inouhe and Yamamoto (1991). More recently, biochemical studies were performed about the toxic effect of external galactose on Arabidopsis roots (Althammer et al. 2020, 2022; see below).

Galactose is not only toxic for plants but also for fungi (e. g. Schuler et al. 2018) and animals (Lai et al. 2009). Humans, who have a genetic disorder to recycle galactose into UDP-galactose develop galactosemia, a disease which may be life threatening for breastfed babies due to the high amount of galactose coming from lactose (Haskovic et al. 2020). This disease has likewise been known for more than 100 years and despite a lot of efforts, the molecular mechanism is not well understood.

However, it has been shown in all model systems investigated so far, that the conversion of galactose to galactose-1P is required for galactose toxicity (Haskovic et al. 2020). In Arabidopsis, a mutant in galactokinase shows no symptoms of galactose toxicity and grows similar to wild type seedlings on plates with sucrose (Egert et al. 2012). The galactokinase mutant accumulates galactose in the cells, because the recycling pathway to UDP-galactose is disrupted (Egert et al. 2012). For yeast it has been suggested, that galactose-1P is an inhibitor of phosphoglucomutase, which would disturb the equilibrium between glucose-1-phosphate and glucose-6-phosphate (Mumma et al. 2008). Recently, Althammer et al. (2020) investigated the inhibition of recombinant Arabidopsis phosphoglucomutase, but neither evidence for the inhibition by galactose-1P, nor for the conversion of galactose-1P to galactose-6P were found.

In view of contradictory biochemical data and considering the fact that surprisingly little is known about the effect of galactose on the fine structure of cells, we selected another approach to shed more light on galactose toxicity in plants. We used various microscopical methods to investigate the effect of galactose on roots of Arabidopsis in more detail. Our study revealed a number of unexpected changes (e. g. incomplete cell wall formation, binuclear cells) when plants were grown on media with low concentrations of galactose (mostly 1 mM). To our knowledge, this is the first time that abundant cell wall stubs have been observed in wild type plants in the absence of either an inhibitor of vesicle transport or a mutation in the vesicle transport machinery (compare Reichardt et al. 2007).

## Material and methods

### Plant material and growth conditions

*Arabidopsis thaliana* (L.) Heynh. wild type seeds were obtained from the Arabidopsis stock centre (N60000; NASC Nottingham). A T-DNA insertion line (GabiKat_489_D10) in the gene for galactokinase (At3g06580) was used as knock out (*galk*), having no galactokinase activity. A *WOX5:GFP* reporter line for detecting root quiescent center cells was kindly obtained from Thomas Laux, Freiburg, Germany. *SCARECROW:YFP* (*SCR:YFP*; NASC N2106124) and *SHORTROOT:YFP* (*SHR:YFP*; NASC N2106109) reporter lines were obtained from the Arabidopsis stock centre.

Seeds were surface sterilized by ethanol for 5 min and germinated on 0.5 × MS plates (Basal Salt Mixture, Duchefa #M0245, Haarlem, Netherlands), pH 5.7 (KOH) containing 0.8% plant agar (Duchefa) and 0.5–5 mM galactose or sucrose. This galactose or sucrose-containing standard medium was modified in different ways: (i) Supplementation with 100 and 200 mM mannitol or with 20 fold higher amount of boric acid compared to standard MS plates. (ii) Replacement of MS by Knop, pH 6.4 (KOH) (Sijmons et al. 1991). (iii) Replacement of plant agar by either 0.8% phytagel (Sigma-Aldrich, Vienna, Austria) or 0.4% gelrite (Duchefa). Plants were grown in a growth chamber under short-day conditions at 23 °C with 10 h light.

The times (days) given in the experiments refer to day zero when the Arabidopsis seeds sown on agar plates were transferred to the growth chamber.

All experiments were performed at least three times.

### Light microscopy

Light microscopy images were taken with a Leica MZFLIII (Leica microsystems) using a Leica DFC 320 camera and a Leitz Dialux 20 equipped with an Olympus EOS 7D camera. For lignin staining, roots were treated with phloroglucinol (Merck, Darmstadt, Germany; 1 g phloroglucinol in 40 ml 92% ethanol) for about 2 min, transferred to concentrated hydrochloric acid and investigated immediately.

### Confocal laser scanning microscopy

For *in-vivo* staining of the plasma membrane, intact roots were stained with red fluorescent FM4-64 [N-(3-triethylammoniumpropyl)-4-(6-(4-(diethylamino)phenyl)hexatrienyl) pyridinium dibromide] or with green fluorescent FM 1-43FX [N-(3-triethylammoniumpropyl)-4-(dibutylamino)styryl pyridinium dibromide], both from Thermo Fischer (Vienna, Austria). The dyes were used at a concentration of 10 μM diluted from a 100 μM (FM4-64) or 500 µM (FM1-43FX) stock solution in artificial fresh water (1 mM CaCl_2_, 0.1 mM NaCl, 0.1 mM KCl). Roots were pulse labelled for 2 min and washed with distilled water before examination.

Nuclei of root tip cells were labelled with DAPI (4′,6-diamidino-2-phenylindole; Roth, Karlsruhe, Germany). The working solution (2 µg/ml) was diluted in distilled water from a 100 µg/ml stock solution in distilled water. Intact roots were stained for 10 min and washed in distilled water.

The confocal imaging was performed on a Leica TCS SP5 laser scanning microscope attached to an inverted microscope (Leica Microsystems). For image acquisition a 25 × water immersion objective with a numerical aperture of 0.95 was mainly used. All images included in this study are single optical sections.

### Electron microscopy

Roots of seedlings were cryofixed in a Leica EMPACT high-pressure freezer and freeze substituted in a Leica EM AFS freeze-substitution apparatus (Leica Microsystems).

Substitution was performed at −80 °C in acetone containing 2% OsO_4_ and 0.05% uranyl acetate for 60 h. The temperature was raised to −30 °C within 4 h, to −20 °C within 10 h and then brought to room temperature. After washing three times in acetone, the roots were transferred into propylene oxide and embedded stepwise in Agar low viscosity resin (Agar Scientific, Essex, Great Britain).

Thin sections (Leica EM UC7; Leica Microsystems) were stained with uranyl acetate and lead citrate. Micrographs were taken at elastic brightfield mode with a LEO 912 transmission electron microscope equipped with an in-column energy filter (Zeiss, Oberkochen, Germany). Figures were prepared using GIMP (https://www.gimp.org/) and Microsoft Powerpoint (https://www.microsoft.com).

## Results

### Galactose toxicity stops root growth and requires galactokinase activity

This study investigated morphological changes in roots of *Arabidopsis thaliana* wild type Col 0, grown on MS-agar plates containing 1 mM galactose (most experiments). Wild type seedlings grown on MS-agar plates with 1 mM sucrose were used as reference. Galactokinase (*galk*) knockout plants, that are not sensitive to galactose (Egert et al. 2012), also served as controls.

**Fig. 1:**
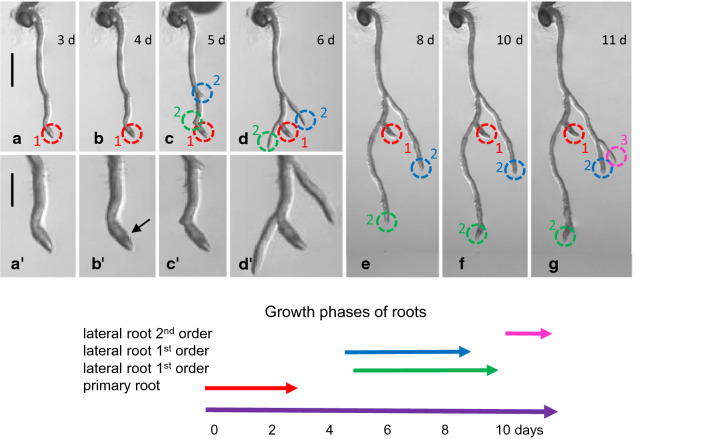
Characterization of the galactose toxicity phenotype (root morphology). **a**–**g** Seedlings grown on 1 mM galactose for 11 days (d). The primary root (red circle) stops growing after three days. The root tip thickens and dark structures (arrow in **b’**) appear (**a’–d’**). Lateral roots develop on day 5 (blue and green circle) close to the tip of the primary root and overgrow the primary root. By day 10 both lateral roots have stopped elongation. On day 11, a new lateral root (pink circle) emerges from the blue circled lateral root. Bars 1 mm (**a–g**) and 0.5 mm (**a’–d’**)

During our studies on nucleotide sugar recycling pathways, we noticed a strong change in Arabidopsis root morphology when seeds were germinated on MS-agar plates with a low concentration (3 mM) of galactose (Althammer et al. 2020). The galactose toxicity phenotype was characterized by root growth inhibition and severe alterations in root morphology. Figure 1a–g shows the root growth of a seedling on a plate containing 1 mM galactose, observed over 11 days. The primary root (red circle) stopped growing after three days. The root tip thickened and dark structures appeared on day 4 (Fig. 1a’-d’). Lateral roots developed on day 5 (blue and green circle) close to the tip of the primary root and overgrew the primary root. By day 10 both lateral roots stopped elongating. On day 11, a new lateral root (pink circle) emerged from the blue circled lateral root. This cascading growth pattern is repeated every few days, depending on the galactose concentration in the medium. Suppl. Fig. S1a and b show a comparison of seedlings grown on 1 mM sucrose and on 1 mM galactose for 11 days. A root grown on 1 mM galactose for 21 days formed lateral roots of 4th order (Suppl. Fig. S1c).

The lower the galactose concentration, the later the effects occurred (Suppl. Fig. S2). Only about 50% of the wild type roots showed modifications after one-week growth on 0.5 mM galactose (Suppl. Fig. S2a-c). On the other hand, 3 mM galactose in the MS medium affected all plants and morphological changes were already observed after 3 days (Suppl. Fig. S2d).

**Fig. 2:**
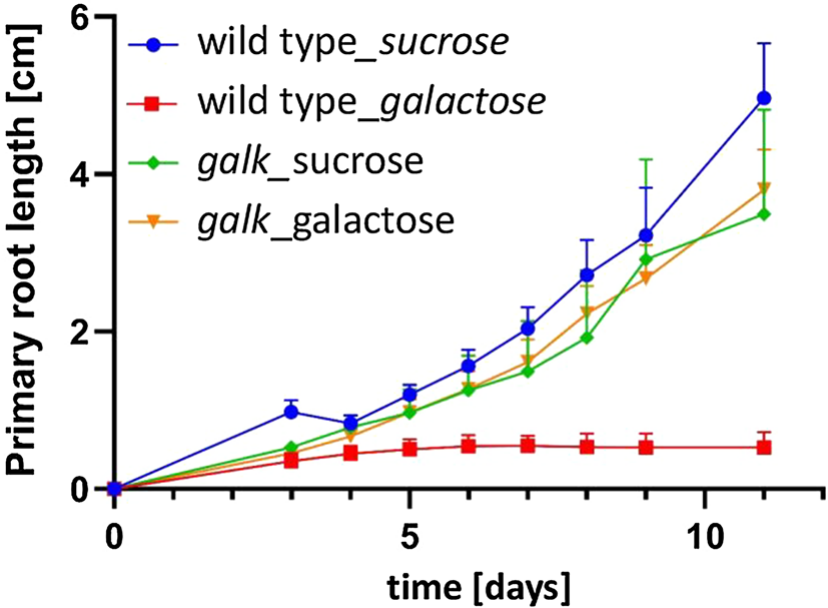
Characterization of the galactose toxicity phenotype (root elongation). Primary root lengths of *Arabidopsis thaliana* seedlings (wild type and *galk*) grown on 0.5 × MS plates supplemented with 1 mM sucrose versus 1 mM galactose (*n* = 12 seedlings; data are means ± SD)

**Fig. 3:**
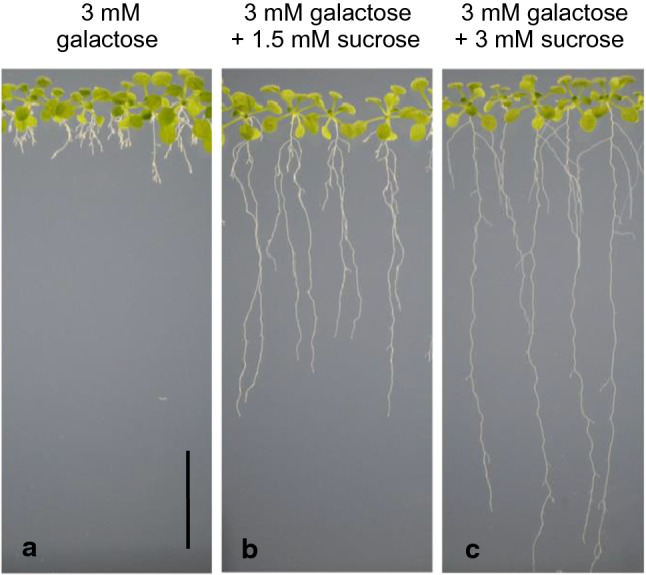
Sucrose suppresses the galactose toxicity phenotype. Seedlings of *Arabidopsis thaliana* wild type grown for 3 weeks on 3 mM galactose (**a**), 3 mM galactose + 1.5 mM sucrose (**b**) and 3 mM galactose + 3 mM sucrose (**c**). Bar 2 cm

As described above, the first obvious effect of 1 mM galactose on *Arabidopsis thaliana* wild type seedlings was the inhibition of primary root growth after 3–4 days (Fig. 2), whereas in controls the primary roots elongated continuously during an observation period of eleven days (Suppl. Fig. S1). Time-dependent elongation of primary roots in *galk* plants grown on galactose or on sucrose plates was similar to root length increase in the wild type on sucrose plates (Fig. 2). The addition of sucrose to galactose plates suppressed the galactose toxicity phenotype in a dose-dependent manner (Fig. 3). The almost complete disappearance of the galactose toxicity phenotype on plates with sucrose may be the reason why galactose toxicity has not yet been observed in other studies since growth media are often supplemented with sucrose (Wang et al. 2015). In contrast to roots, the morphology of the shoots was not affected (Suppl. Fig. S1d, e). Sucrose synthesized in leaves during photosynthesis might have prevented the occurrence of a galactose toxicity phenotype in shoots. The galactose toxicity phenotype could also be observed when roots grown on sucrose were transplanted onto galactose containing plates. First signs of the galactose phenotype were visible 1 to 2 days after transferring plants grown for 5 days on MS-agar with 1 mM sucrose onto plates with 1 mM galactose (Suppl. Fig. S2e, f). For further studies, we only used plants grown on galactose containing media, to omit an additional manipulation step.

### Galactose increases root tip diameter and causes lignification

**Fig. 4:**
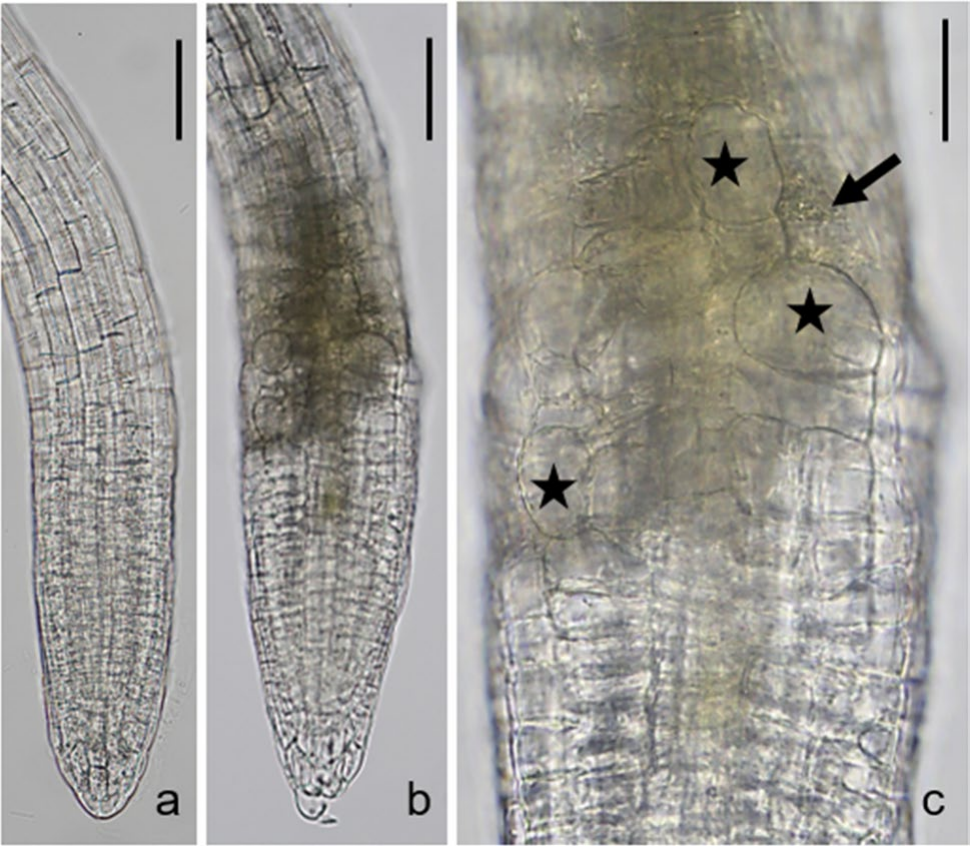
Rhizodermal cell bulging. Bright-field images of primary roots of *Arabidopsis thaliana galk* (**a**) and wild type (**b, c**) both grown on 1 mM galactose for 6 days. The diameter of wild type primary root is increased compared to that of *galk*. Note abnormally large rhizodermal cells (asterisks in **c**) and slightly opaque areas consisting of at least some dead cells (arrow in **c**) in the wild type root. Bars 100 µm (**a**, **b**), 30 µm (**c**)

**Fig. 5:**
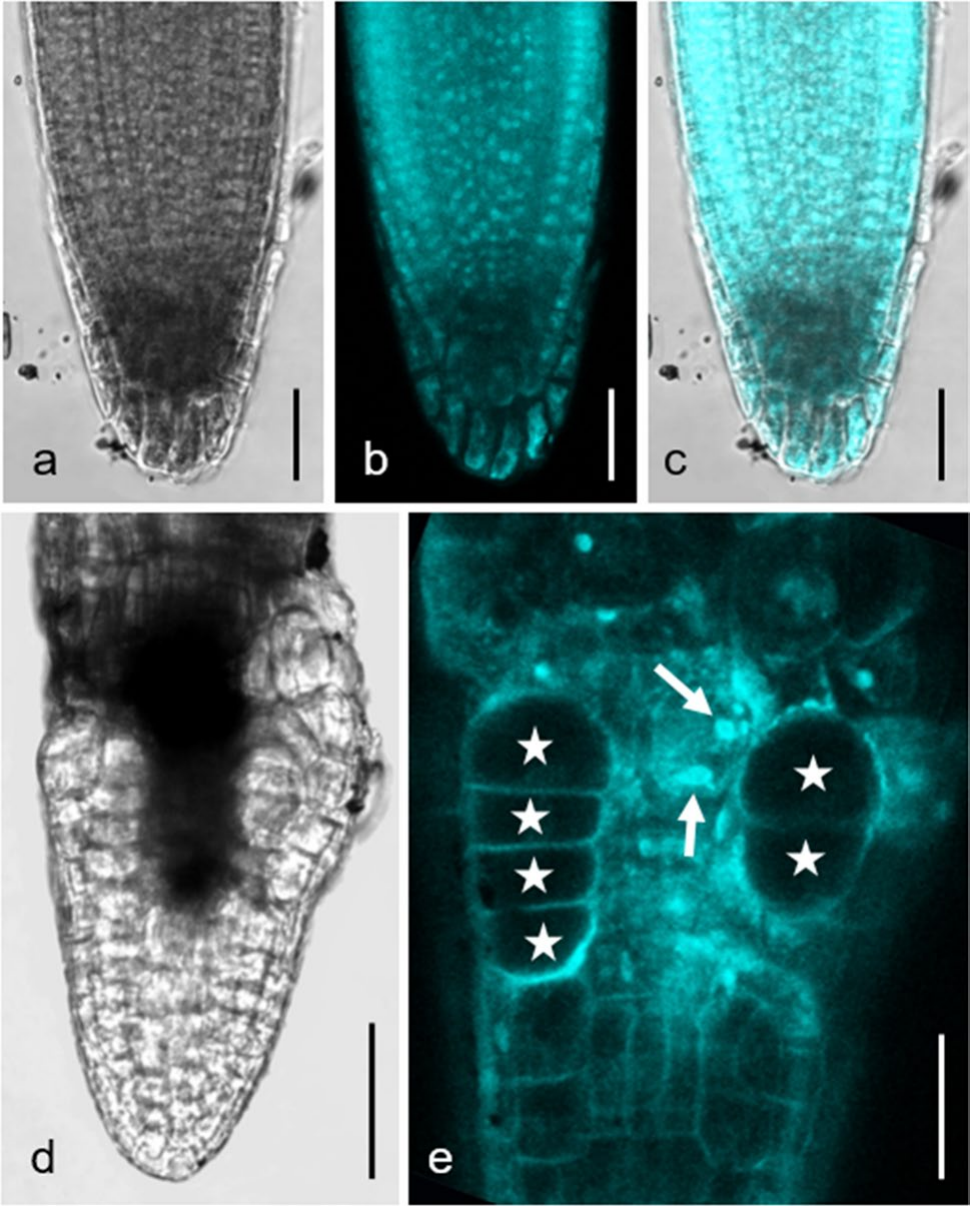
Rhizodermal cell bulging and DAPI staining. *Arabidopsis thaliana* wild type grown on 5 mM sucrose (**a**–**c**) and 5 mM galactose (**d, e**) for 3 days. Asterisks in **e** indicate enlarged rhizodermal cells, the arrows point to two nuclei within a single cell. Note irregular size and shape of nuclei in galactose treated root as compared to the control. **a** and **d** Bright field images. **b** and **e** Fluorescence images. **c** Merged image. Bars 25 µm (**a**–**c**, **e**) and 50 µm (**d**)

**Fig. 6:**
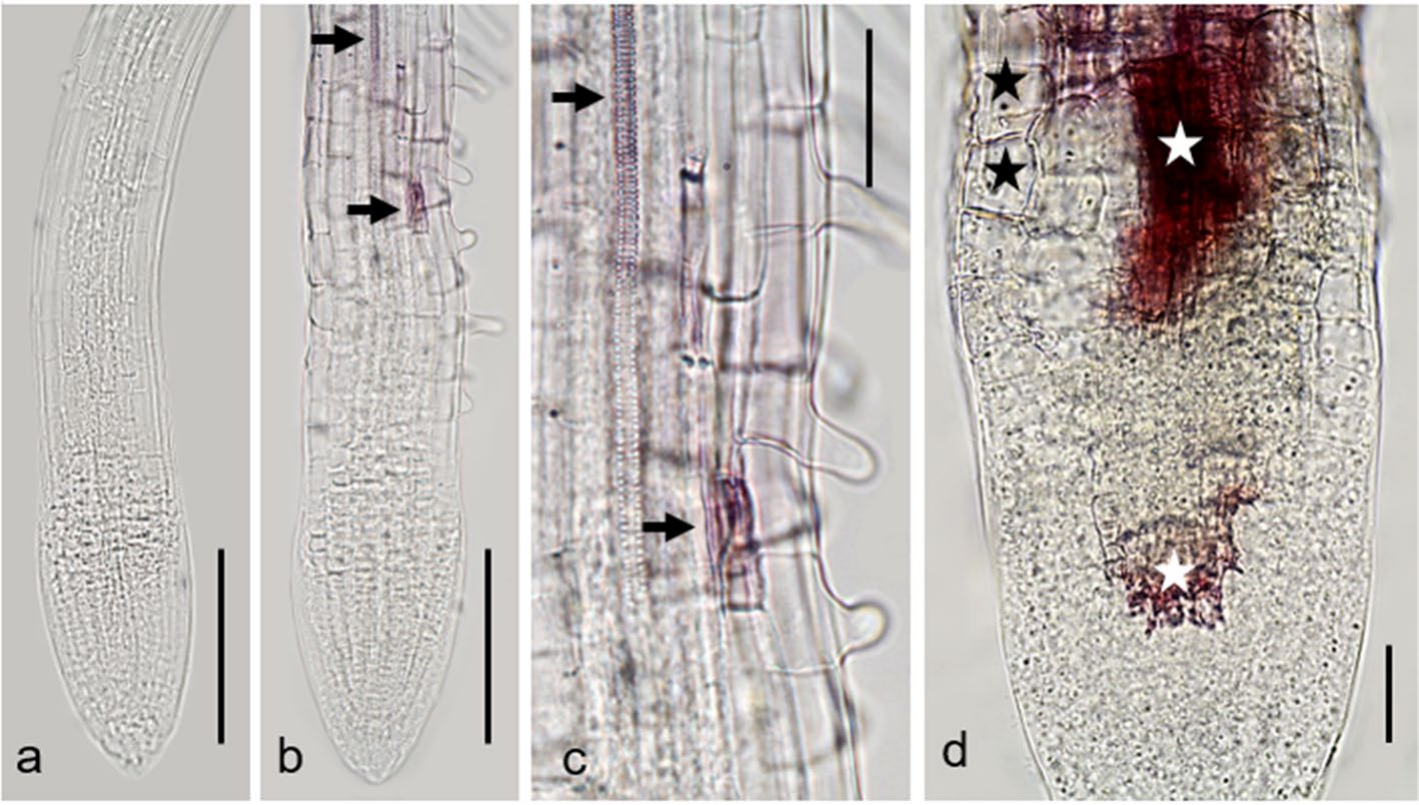
Lignification revealed by phloroglucinol staining. *Arabidopsis thaliana* wild type primary roots were grown for 4 days on 1 mM sucrose (**a**), for 4 days on 1 mM galactose (**b, c**) and for 5 days on 1 mM galactose (**d**). **a** No labelling is visible in the control on sucrose. **b** and **c** After 4 days of galactose the xylem (upper arrow) is stained by phloroglucinol (purple) already 500 µm from the very tip. A single cell (lower arrow) in the endodermis/cortex is also stained. **c** is an enlarged detail of **b**. **d** After 5 days of growth on galactose larger areas in the root tip are labelled (white asterisks). Black asterisks mark enlarged rhizodermal cells. Bars 150 µM (**a, b**), 75 µm (**c**) and 30 µm (**d**)

**Fig. 7:**
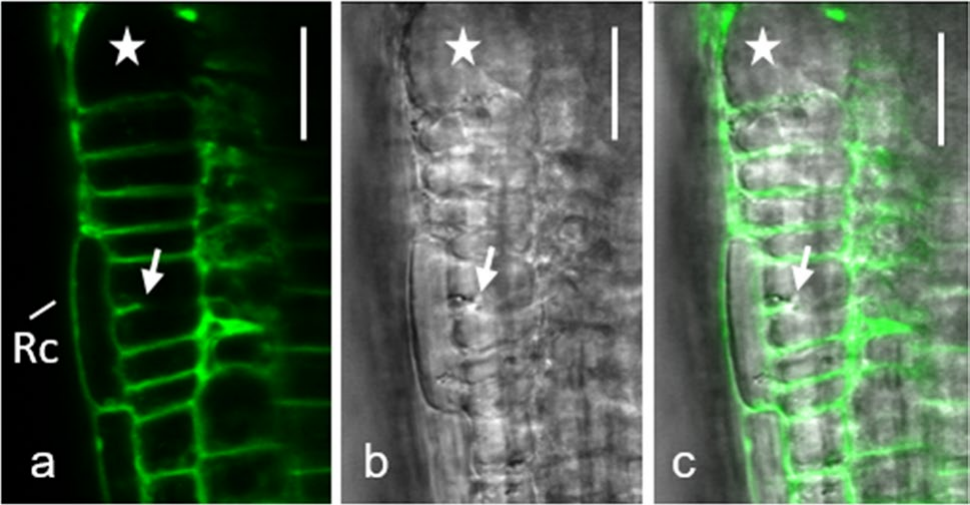
Cell wall stub. Primary root of *Arabidopsis thaliana* wild type grown on 3 mM galactose for 6 days and stained with FM 1–43. A cell wall stub (arrow) is clearly visible in a rhizodermal cell. An enlarged cell is marked by an asterisk. Rc (root cap). **a** Fluorescence image. **b** Corresponding bright-field image. **c** Merged image. Bar 20 µm

Light microscopy inspection revealed that, as compared to controls, root tips of wild type seedlings grown on galactose plates increased in diameter (Fig. 4) and the regular arrangement of cells disappeared so that sometimes cells could not be assigned to a distinct tissue (Suppl. Fig. S3a, b). Slightly opaque structures appear in the root tip (Fig. 4b). The contracted cytoplasm with coarse granular content and the absence of cytoplasmic streaming suggested that dead cells contributed to the composition of the dark structures (Fig. 4c). The development of enlarged rhizodermal cells was also characteristic for root tips grown on galactose plates. They were often arranged in files and became larger, the farther they were from the very tip (Fig. 4c; compare Figs. 5d, e, 6d, 7a–c).

**Fig. 8:**
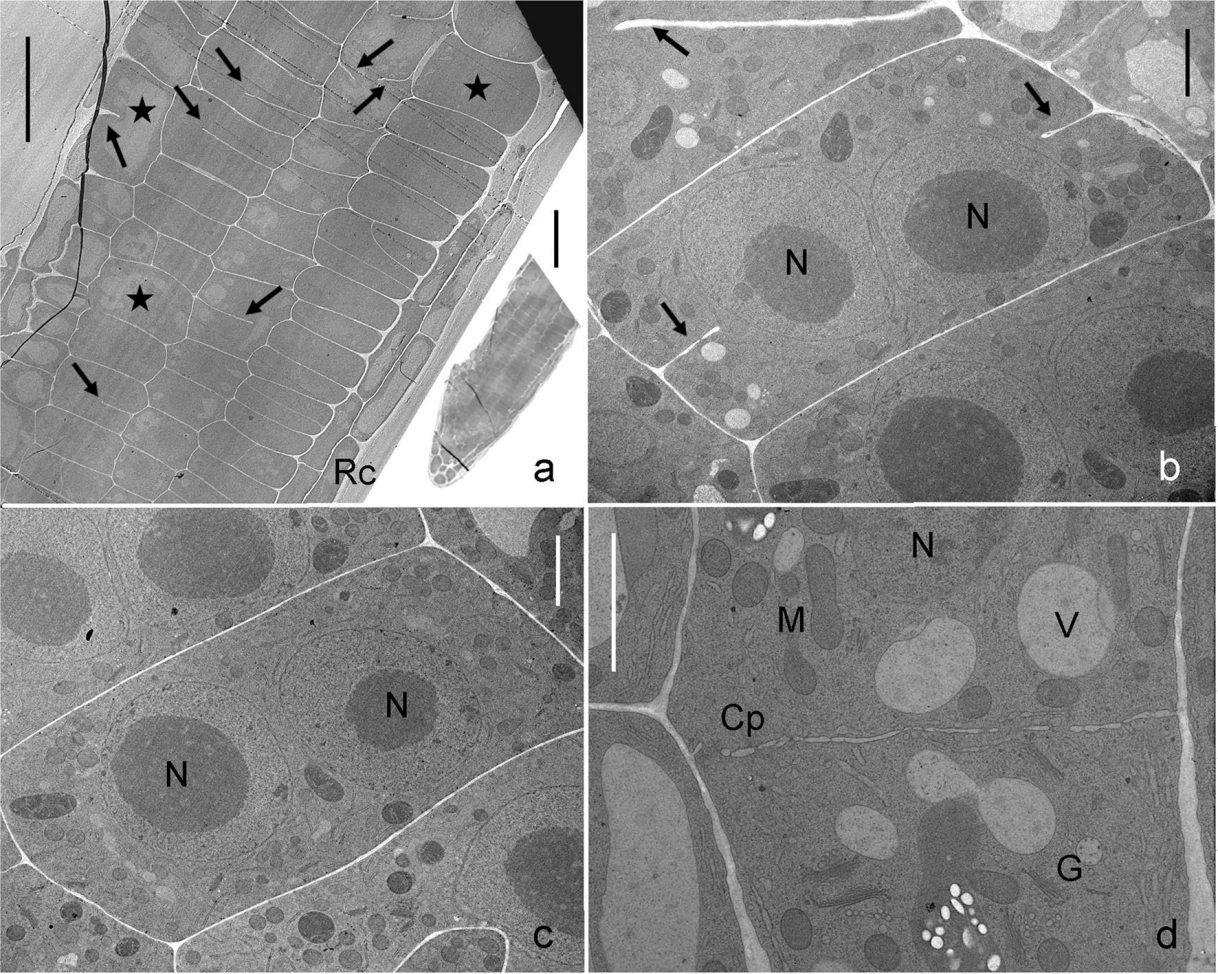
Cell wall stubs. Electron microscopical images of *Arabidopsis thaliana* wild type roots grown on 1 mM galactose (**a**–**c**) and primary root grown on 1 mM sucrose (**d**) for 7 days. **a** Cell files in a longitudinal section (the whole section is shown in the inset) of a primary root tip. Numerous cell wall stubs (black arrows) and some enlarged cells (asterisks) are visible. **b** and **c** Binuclear cells of a lateral root with (arrows in **b**) or without (**c**) cell wall stubs in detail. **d** Forming of a cell plate (Cp) in a control cell. G, Golgi body; M, mitochondrium; N, nucleus; Rc, root cap; V, vacuole. Bars 50 µm (inset in **a**), 20 µm (**a**), 2 µm (**b**–**d**)

Figure 5a–c shows root tips of Arabidopsis wild type grown on sucrose. The DAPI staining revealed the typical size and roundish shape of nuclei in meristem cells. Figure 5d, e shows root tips of seedlings grown on 5 mM galactose. Note that galactose induced morphological changes occurred much earlier (after 3 days) and were more severe than in seedlings grown on 1 mM galactose (Fig. 4). The nuclei often had irregular sizes and shapes (Fig. 5e), indicating premature differentiation. Furthermore, we occasionally observed two nuclei per cell (arrows in Fig. 5e; compare Fig. 8b, c).

To get insight into the nature of the slightly opaque areas arising from galactose treatment, different staining methods were used. Attempts to stain living cells with FDA (fluorescein-diacetate) or to label the nuclei of dead cells with propidium iodide inside the dark structures failed, as both dyes seem not to penetrate the dark structure (data not shown). We thus focused on phloroglucinol, a standard stain for lignin which, under acidic conditions, specifically labels O-4-linked coniferyl and sinapyl aldehydes in lignifying cell walls (Pomar et al. 2002; Kapp et al. 2015). Roots of controls (Fig. 6a) were not stained by phloroglucinol, with exception of older parts of the xylem (not shown). In wild type roots grown on galactose plates, phloroglucinol reveals at least partial lignification of the dark structures (Fig. 6b–d). The xylem of galactose treated roots was stained by phloroglucinol far closer to the tip than the xylem in controls (Fig. 6b, c), consistent with cessation of root growth. Furthermore, the lignified cells are not limited to xylem vessels but often occurred in irregular clusters including the cortex (Fig. 6d; compare Suppl. Fig. S4e, f, Suppl. Fig. S8).

An increase in root tip diameter has also been reported for roots grown on media containing submillimolar concentrations of mannose (Baskin et al. 2001). Suppl. Fig. S4 shows that roots tips grown on mannose were straighter and shorter than those grown on galactose. Lignification was restricted to the central cylinder and the endodermis, and it was absent from the very tip. Furthermore, enlarged rhizodermal cells (see below) were not found. The toxicity of mannose is associated with phosphorylation at C6 (Baskin et al. 2001). In contrast, we found no evidence that Gal-6-phosphate is accumulating in plants (Althammer et al. 2020). Therefore, galactose toxicity is clearly different from mannose toxicity.

### Some cells enlarge strongly, form cell wall stubs and become binuclear

As shown above, the development of enlarged rhizodermal cells was characteristic of root tips grown on galactose plates. Some of these cells were characterized by incomplete cell divisions (Figs. 7, 8; compare Suppl. Fig. S3c, d). Cell wall stubs develop in some cells, other cells contain two nuclei (Fig. 8a–c; compare Fig. 5e). Formation of cell wall stubs was not restricted to the rhizodermis but was also found in other tissues (see cross-section in Suppl. Fig. S5a–c). Apart from the existence of two nuclei with or without cell wall stubs, the fine structure of the cells, i. e. the morphology and distribution of organelles, seemed to be quite unchanged (Fig. 8b, c). Occasionally, microtubules could be seen along the plasma membrane of cell wall stubs (Suppl. Fig. S5c, d). Figure 8d shows a typical cell plate forming in a control root grown on sucrose. Cytoplasmic streaming was often observed in cells with incomplete walls, suggesting that the actin cytoskeleton remained more or less intact, at least for a while. Suppl. Fig. S6 shows that the shape and size of the cell wall stub remained unchanged over an observation period of 100 min.

### Files of cells degenerate on galactose media

**Fig. 9:**
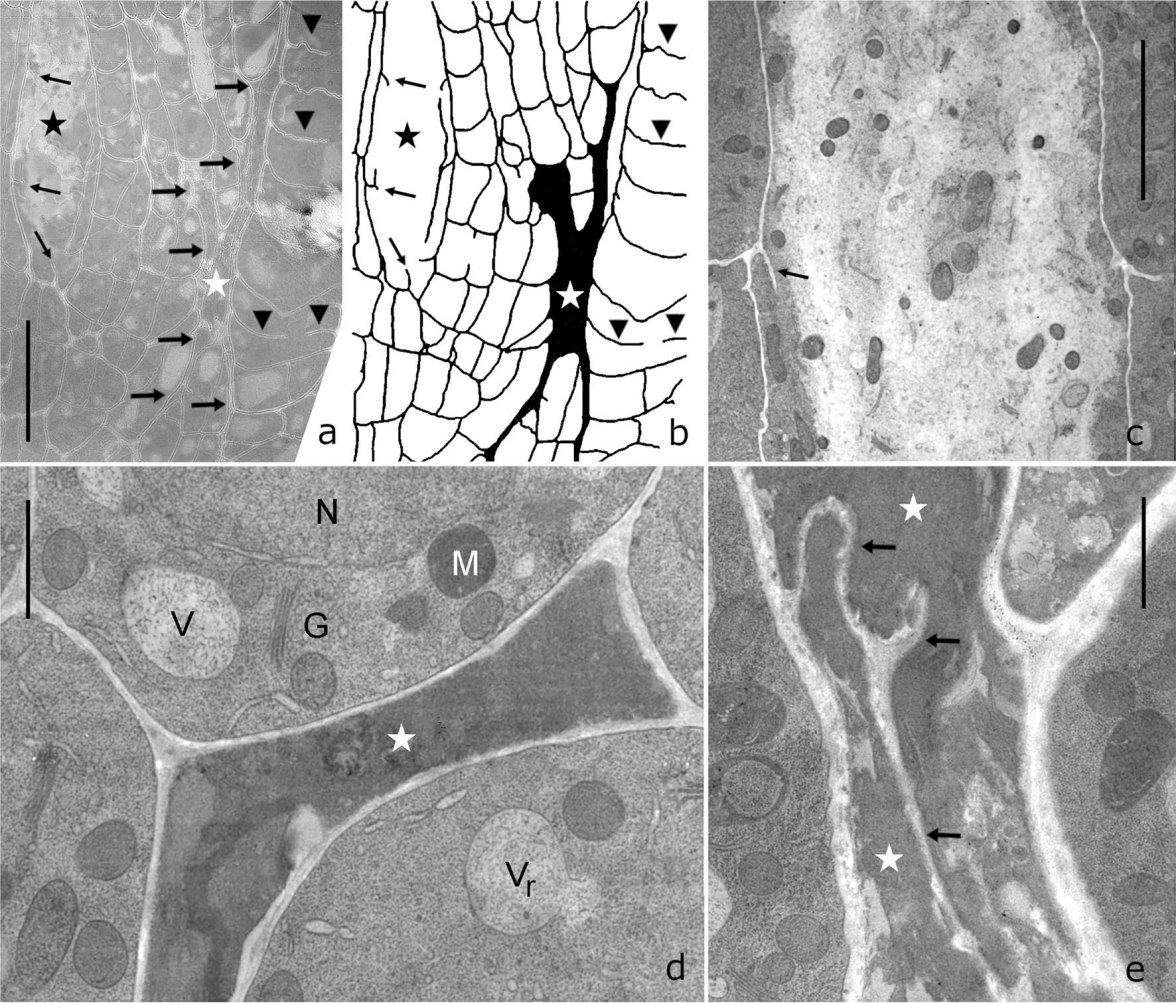
Degenerated cells. Electron microscopical images of *Arabidopsis thaliana* wild type primary roots grown on 1 mM galactose for 7 days. **a** Overview of a longitudinal section through the root tip, outlines of cell walls are drawn in (**b**). Files of cells with cell wall stubs and/or destroyed cell walls (thin arrows) are marked by a black asterisk. Long files of squeezed degenerated cells are marked by thick arrows in (**a**) and are black coloured in (**b**). The white asterisk indicates a fusion site of cell files with degenerated cytoplasm (black area in **b**; compare **e**). Arrowheads mark cell wall stubs in files of cells with intact cytoplasm at the right side of (**a**) and (**b**). **c** Loss of tonoplast indicates an early stage of cytoplasm degradation. Scattered organelles can still be identified, the arrow indicates a cell wall stub. The image corresponds to the area indicated by a black asterisk in (**a**) and (**b**). **d** and **e** Degenerated cells (white asterisks) without discernable organelles. Cell wall remnants are seen in **e** (arrows). Note healthy-looking cytoplasm in the surrounding cells. However, a ruptured tonoplast (V_r_) in (**d**) indicates an early sign of damage. *G *Golgi body, *M* mitochondrium, *N* nucleus, *V* vacuole. Bars 20 µm (**a, b**), 4 µm (**c**) and 1 µm (**d, e**)

Figure 9 shows electron micrographs of wild type seedlings grown on galactose for 7 days. On the same longitudinal section of a root tip, healthy-looking cells with clearly identifiable organelles can be seen along with files of cells with aberrant cell walls and degenerated cytoplasm (black asterisks in Fig. 9a, b). Cross walls were either incomplete (cell wall stubs; see above) or ruptured (see thin arrows in Fig. 9a–c). Files of damaged cells that look squeezed (asterisk in Suppl. Fig. S5e) formed tubular structures within the root tissue (Fig. 9, thick black arrows in a and black area in b). These files could be more than 120 µm long. Several parallel running cell files were observed to lay side by side (asterisk in Suppl. Fig. S5e) or seemed to fuse (white asterisks in Fig. 9a, b). One of the first morphological signs of damage at the cell level was the disappearance of the tonoplast so that the contents of vacuole and cytoplasm mixed (Fig. 9c). Other early signs of damage were the irregular size of nuclei and the disintegration of the nuclear membrane (Fig. 5e; Suppl. Fig. S5e, f). In later stages, a mostly compact mass was located between the cell walls of these degenerated cells where organelles were no longer identifiable (Fig. 9d, e). We assume that dead cells with weakened walls became squeezed by the turgor of the neighbouring cells. Suppl. Fig. S7 shows different stages of cell degeneration.

### Stem cell markers are expressed even after cessation of root growth

**Fig. 10:**
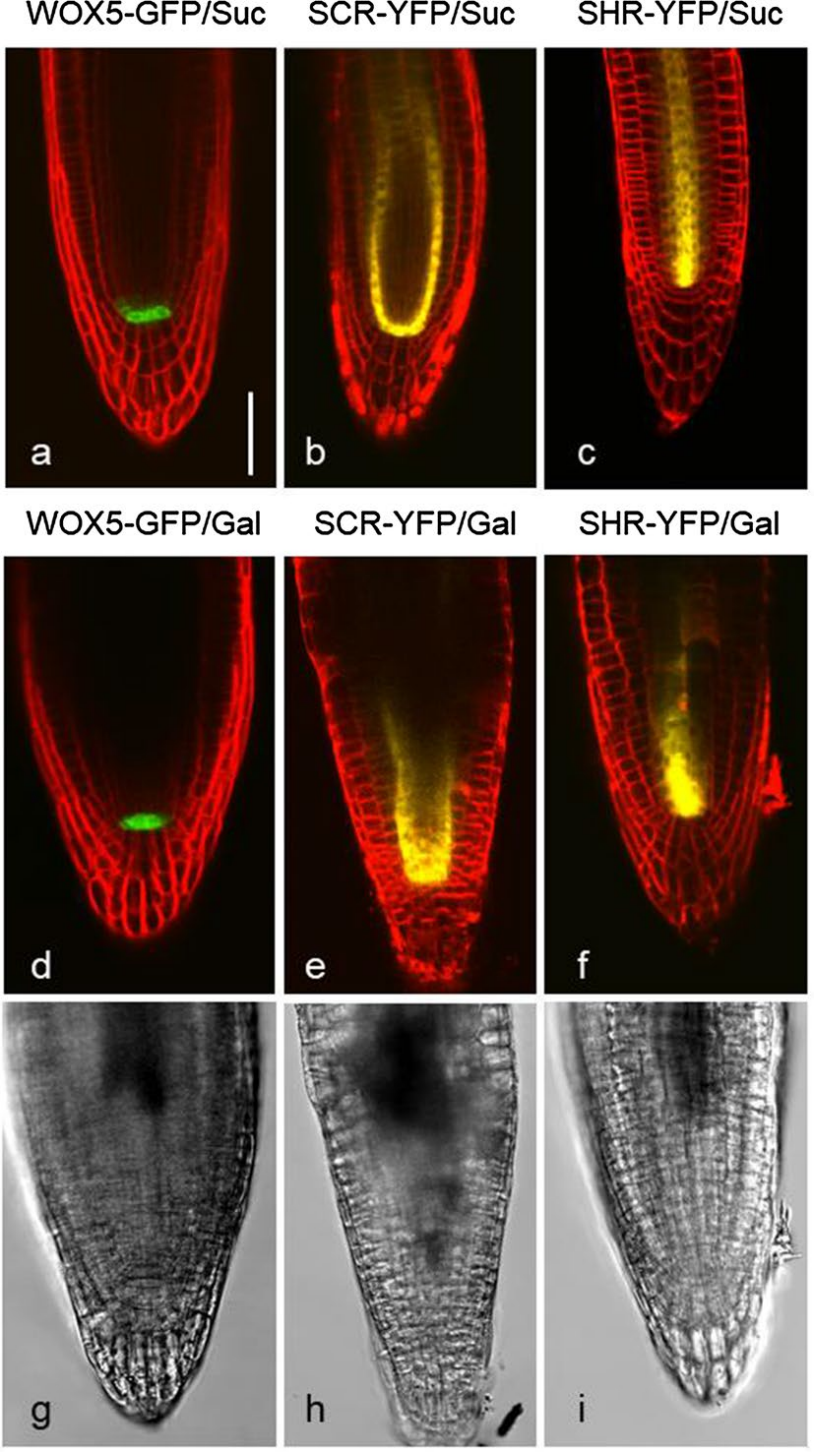
Expression of stem cell markers (*WOX5:GFP* and *SCARECROW:YFP*; *SCR:YFP)* and vasculate marker (*SHORTROOT:YFP; SHR:YFP*). Primary roots of *Arabidopsis thaliana* wild type were grown on 1 mM sucrose (Suc; **a**–**c**) and on 1 mM galactose (Gal; **d**–**i**) for 4 (**a, d, g**) and 5 (**b, c, e, f, h, i**) days, respectively. The markers are well expressed despite significant changes in root tip morphology. **a**–**f** Fluorescence of markers and FM4-64 (merged images), **g**–**i** Bright field images corresponding to (**d**–**f**). Bar in **a** for all images 50 µm

To test whether galactose impaired the function of root stem cells, different cell-type specific reporter gene lines were grown on sucrose and galactose plates and then investigated with confocal microscopy (Fig. 10). We used *WOX5:GFP* (Fig. 10a, d, g; expressed in the quiescent centre), *SCARECROW (SCR:YFP*; Fig. 10b, e, h; expressed in the quiescent centre and the endodermis) and *SHORTROOT (SHR:YFP*; Fig. 10c, f, i; expressed in the stele), which play a role in controlling the function of the stem cells of the root meristem. The different stem cell markers were all well expressed after several days of growth on galactose plates (Fig. 10d–f), even when the morphological changes of the root tip were already distinct (Fig. 10g–i). A similar observation was recently described for *folylpolyglutamate synthetase1* mutants with a defect in folate C1-metabolism (Reyes-Hernandez et al. 2014). Although the roots were short and branched and only very slowly growing, a number of stem cell markers was still clearly expressed. From these observations, we conclude that the stem cells in seedlings grown on galactose plates were not affected by galactose in the early stages, and therefore were not the primary cause of galactose toxicity.

### The galactose toxicity phenotype is similar on different media

We tested the robustness of the galactose toxicity phenotype by comparing two plant growth media and two alternative solidifying polymers (Suppl. Fig. S8). Apart from MS-medium, which is the standard growth medium for Arabidopsis, we also tested Knop-medium. Both media with galactose lead to the same phenotype, indicating that the different ion concentration in MS- or Knop-media was not important for galactose toxicity (Suppl. Fig. S8a, b). We replaced the commonly used plant agar with gelrite or phytagel as solidifying agents, but again the root morphology remained the same on all media (Suppl. Fig. S8c-f). We also used a 20-fold higher boron concentration in our standard MS-media, to allow crosslinking of rhamnogalacturonanII pectic polymers. Again, high boron was without effect on root development (Suppl. Fig. S8g, h). It has been reported for maize roots, that galactose causes rapid changes in the osmotic pressure of root cells (Pritchard et al. 2004). The addition of 100 mM or 200 mM mannitol to galactose-MS-agar plates was without obvious effect on root morphology (Suppl. Fig. S8i–l). Taken together, these variations of growth conditions had no effect on the root morphology confirming the robustness of the galactose toxicity phenotype.

Arabinose has been reported to be toxic for a plant mutant with a point mutation in the arabinokinase gene (Dolezal and Cobbett 1991; Behmuller et al. 2016). However, wild type seedlings grown on MS-plates with 3 mM arabinose grew normally in comparison with wild type seedlings on galactose plates. Thus, the disturbance of root development was specific to galactose.

## Discussion

### Galactose toxicity depends on galactokinase activity

Here we analysed the major changes in root morphology of Arabidopsis seedlings grown on MS-medium with low (mostly 1 mM) galactose concentration. The most obvious effect of exogenous galactose is that primary roots stop growing within a few days. Lateral roots emerge, overgrow the primary root, but likewise stop growing after a couple of days. This cascading growth pattern repeats every few days and leads to the formation of bushy branched roots. The root tips are characterized by a slightly opaque, dark region, by enlarged cells with or without cell wall stubs and by heavily lignified cell walls. As shown in Fig. 2, the phenotype depends on the activity of galactokinase as the first enzyme of galactose recycling to UDP-galactose.

### Exogenous galactose induces cell wall stubs in wild type plants

A striking phenotype of galactose toxicity is the occurrence of cell wall stubs. Cell plate assembly occurs through the cooperation of the post-Golgi endomembrane system and the phragmoplast, which consists of a bipolar array of microtubules and actin filaments (Smertenko et al. 2017). The concept of cell plate formation was challenged by the finding that cell plates are not always centred during cytokinesis, but attach preferentially to one side of the parental cell wall. The attached cell plate then elongates towards the other side of the cell (“polarized cytokinesis”, Cutler and Ehrhardt 2002; Bolte et al. 2004).

However, we often see cell wall stubs on both sides of the parental cell and, furthermore, we never observed that cell wall stubs extended towards the other side of the cell and completed cell plate formation. In addition, binuclear cells without any cell wall stubs are found which argues against the hypothesis of “polarized cell division”.

### Cell wall stubs are observed in vesicle trafficking and cell wall mutants and in roots treated with appropriate inhibitors

Cell wall stubs are incomplete cell plates and have been described for a number of mutants defective in vesicle formation, transport, docking or fusion to target membranes. A mutant in the syntaxin *KNOLLE* is such an example (Lauber et al. 1997), and a number of other cytokinesis mutants with a defect in vesicle trafficking are known, including *keule* and *hinkel* (Assaad et al. 1996; Sollner et al. 2002). The inactivation of ARF-GEFs *BIG1–BIG4* which causes the redirection of vesicles from the cell plate to the plasma membrane is another example (Richter et al. 2014). Cell wall stubs and/or radial swelling are also characteristic of cell wall mutants such as *korrigan* (Zuo et al. 2000), *procuste* (Fagard et al. 2000), *rsw1* (*radially swollen 1*, Baskin et al. 1992; Sollner et al. 2002) and *cyt1 (cytokinesis defective1*, Nickle and Meinke 1998). Gu et al. (2016) show that the unstable protein CSLD5, a member of the cellulose synthase-like D-family, participates in the construction of the newly forming cell wall and is rapidly degraded upon completion of cell division. The loss of this cell wall biosynthesis enzyme causes the formation of incomplete cell plates as shown in *csld5* mutants (Gu et al. 2016).

Formation of cell wall stubs can also be induced by inhibitors interfering with membrane trafficking. Reichardt et al. (2007) found that treatment of Arabidopsis wild type roots with concanamycin A, an inhibitor of trafficking at the trans-Golgi network, induces the formation of binuclear cells and cell wall stubs. A similar effect was observed when *gnl1* (*gnom-like1*; a mutant not resistant to brefeldin A) was treated with brefeldin A which interferes with ER-Golgi traffic (Reichardt et al. 2007).

These data suggest that the galactose induced cell wall stubs described in this study are possibly due to disturbed vesicle trafficking or cell wall biogenesis. Indeed, Wang et al. (2015) describe an EMS-mutant, encoding an allele of UDP-glucose-4-epimerase, which has a disturbed vesicle transport in the trans-Golgi network and early endosomes (Uehara et al. 2014; Wang et al. 2015). Interestingly, the mutant phenotype could be complemented by adding low amounts of external galactose. This suggests to us that the level of UDP-galactose must be within a certain concentration range leading to proper galactosylation, whereas conditions of lower galactosylation (Wang et al. 2015) or over-galactosylation, as found after galactose feeding, cause a disturbance in vesicle traffic and/or early endosome organization. Unfortunately, the mechanism for the putative disturbance of vesicle transport during root exposure to galactose containing media is currently unknown (Uehara et al. 2014) and further studies are required.

### Galactose leads to lignified root tips

We see a strong lignin staining with phloroglucinol in the root tips of galactose grown seedlings, which is not observed in *galk*-controls. Lignin in the primary root grown on 1 mM galactose becomes prominent after 3–4 days and lignification increases after 5 days. At this time point, the growth of the primary root has already stopped. Similar observations have been described for isoxaben treated Arabidopsis seedlings (Denness et al. 2011). Isoxaben is a cellulose biosynthesis inhibitor and treated plants have less cellulose in their root cell walls. Cell walls with less cellulose are weaker than regular cell walls and the lignification is interpreted as a stiffening mechanism to locally stabilize the damaged cell wall and to prevent the activation of a cell wall damage response (Denness et al. 2011). Melida et al. (2015) applied the cellulose synthase inhibitor dichlobenil to maize cell cultures. Habituated cultures show a high increase in cell wall phenolics as well as ectopic lignification. It is, however, interesting to note that Arabidopsis cell cultures, habituated to isoxaben do not show ectopic lignification. Rather a change in the cell wall polymer composition toward more pectic polysaccharides was observed in the cell cultures treated with isoxaben (Mansfield et al. 2004), suggesting that plants have established independent procedures to cope with the mechanical stress caused by herbicide action. Our observations that some cell walls are deformed in files of damaged cells probably indicate a weakened, non-lignified cell wall, caused by a small increase in galactose content. Whether the lignification process is induced by the death of neighbouring cells remains to be investigated, as well as a phytohormone mediated lignification (Denness et al. 2011), which might be phenocopied in galactose grown seedlings.

### Galactose induces giant rhizodermal cells independently of osmolytes

Roots grown on galactose develop some giant rhizodermal and cortical cells. This phenotype has often been observed in mutant alleles of UDP-glucose-4-epimerase, causing lower concentrations of UDP-galactose (Andeme-Onzighi et al. 2002; Seifert et al. 2002). The reduced activity of the UDP-glucose-4-epimerase leads to a lower galactosylation of arabinogalactan proteins, but also to a change in the expression pattern. In contrast, Althammer et al. (2022) found that roots contain a higher concentration of galactose in the cell wall. It seems possible that, within a certain range, UDP-galactose is beneficial for the plant, but concentrations beyond this range cause changes in polymers and glycoproteins, and ultimately affect the cell wall architecture. The application of (β-D-Glucose)_3_-Yariv to wild type roots, which is interacting and precipitating arabinogalactan proteins, phenocopies the formation of giant cells in the rhizodermis (Ding and Zhu 1997). Whether plant cells recognize precipitated arabinogalactan proteins as a local increase of arabinogalactan proteins at the precipitation site or as an overall lower presence of arabinogalactan proteins in the plant cell wall, is currently unknown.

The number and size of the giant cells in the rhizodermis of seedlings grown on galactose did not change, even if mannitol at 100 or 200 mM was added to reduce the osmotic pressure in the cells. Thus, the formation of giant cells is not caused by excessive turgor in the rhizodermal cells. The galactose toxicity phenotype leads to a cessation in root growth and, visually, the root branching was more pronounced (Fig. 1; Suppl. Fig. S1; compare Fig. 1 in Althammer et al. 2020). In wheat roots treated with PEG8000 as an osmotic stress compound, Ji et al. (2014) observed premature exhaustion of the root apical meristem and increased formation of lateral roots. In our experiments, the root ceases to grow after a few days, but stem cell markers like *WOX5* are still expressed.

### Galactose toxicity and change in metabolites levels

The effect of galactose on the auxin-mediated extension rates of coleoptiles or hypocotyls has previously been investigated in detail (Yamamoto et al. 1988). Extension growth was stronger inhibited in monocots than in eudicots and the reduction in growth rate starts immediately after the application of galactose (Inouhe and Yamamoto 1991). An important difference between these studies is that we do not apply auxin along with galactose. As shown in Fig. 1, the toxic effect of galactose on Arabidopsis roots is not a general inhibition, as seen by Yamamoto et al. (1988). Primary as well as lateral roots grow normally on galactose plates for approximately three days each and stop thereafter. Likewise, the explanation that depletion of UDP-glucose is responsible for galactose mediated growth inhibition (Yamamoto et al. 1988) does not apply to our study. Althammer et al. (2022) do not see a reduction of UDP-glucose, but an increase in UDP-galactose. Furthermore, by using comprehensive microarray polymer profiling to characterize polymers and glycoproteins from roots grown on galactose, Althammer et al. (2022) found a strong signal increase for arabinogalactan proteins and for hydroxyprolin-rich-glycoproteins, indicating an increase in galactosylation. This differs from the UDP-glucose depletion mechanism, as put forward by Yamamoto et al. (1988). Galactose-1-phosphate is suggested by Inouhe and Yamamoto (1991) to inhibit the formation of UDP-glucose. Higher concentrations of galactose-1-phosphate should thus lead to a more severe phenotype. The study by Althammer et al. (2022) shows, however, that lowering this metabolite increases the strength of the galactose toxicity phenotype in Arabidopsis roots. One conclusion could be that several metabolites of galactose recycling contribute to the observed phenotype. Haskovic et al. (2020) conclude in their review about model systems for (animal) galactosemia, that more than one metabolite is likely causing the disease symptoms. Clearly, more research is needed to elucidate the biochemical and molecular basis of galactose toxicity in plants.

#### *Author contribution statement*

MH and IF performed most of the experiments with microscopy. MA contributed to the experiments. RT outlined the study. IF prepared most of the figures. All authors contributed to the writing of the manuscript.

## Supplementary Information

Below is the link to the electronic supplementary material.

Supplementary file1 (PDF 59 KB)

Supplementary file2 (PDF 101 KB)

Supplementary file3 (PDF 65 KB)

Supplementary file4 (PDF 136 KB)

Supplementary file5 (PDF 452 KB)

Supplementary file6 (PDF 280 KB)

Supplementary file7 (PDF 270 KB)

Supplementary file8 (PDF 210 KB)

Supplementary file9 (DOCX 16 KB)

## Data Availability

All data generated or analysed during this study are included in this published article.

## Abbreviation

*galk*: Galactokinase knockout
