# Supplementary material for: Galactose induces formation of cell wall stubs and cell death in Arabidopsis roots
Source: Planta. 2022 Jul 3;256(2):26. doi: 10.1007/s00425-022-03919-x (PMC9250921; doi:10.1007/s00425-022-03919-x)
Supplement: Supplementary file 1 — Supplementary file1 (PDF 59 KB) [file 425_2022_3919_MOESM1_ESM.pdf]

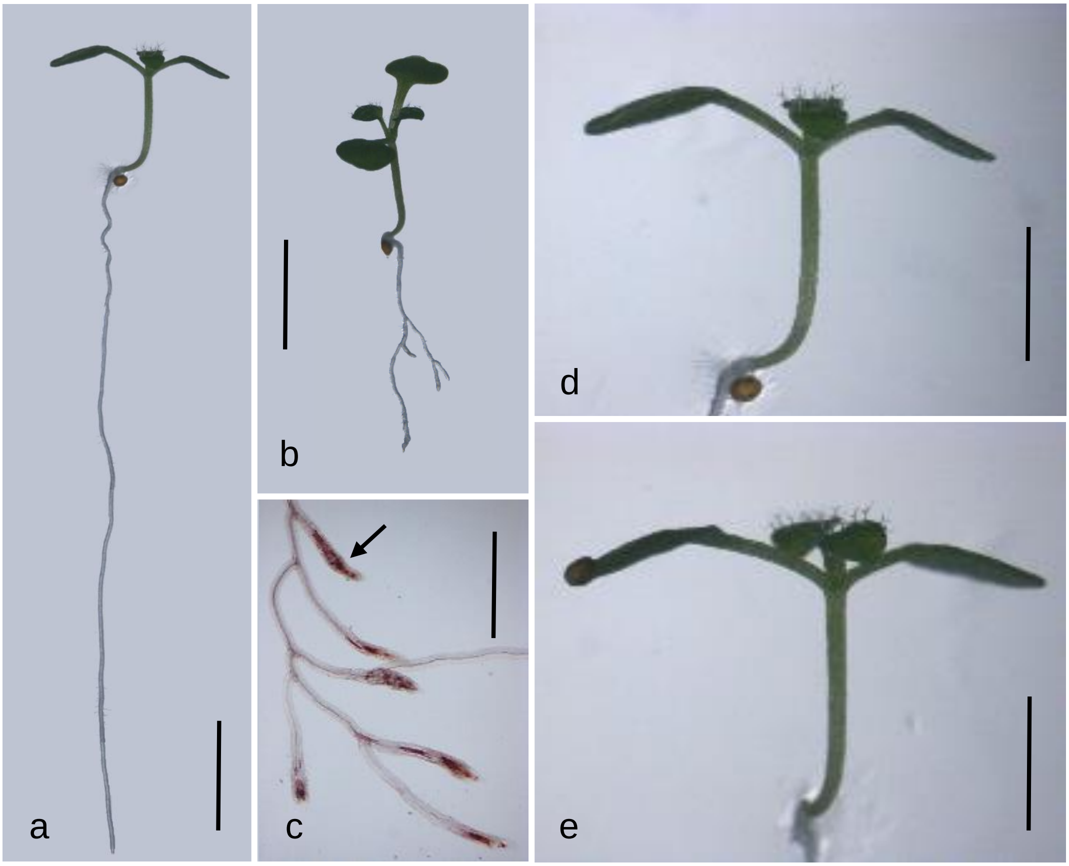

Suppl. Fig. S1 Seedlings of *Arabidopsis thaliana* grown on 1 mM sucrose (**a**, **d**) and on 1 mM galactose (**b**, **c**, **e**) for 11 (**a**, **b**, **d**, **e**) and 21 days (**c**), respectively. Note that the morphology of shoots is not affected by galactose. The root illustrated in **c** was stained for lignin by phloroglucinol. The root tip of the primary root (arrow) and lateral roots up to the fourth order are visible. Note that the root of an 11 day old seedling grown on galactose (**b**) is much shorter and extensively branched in comparison to the root grown on sucrose (**a**). Bars 4 mm (**a**, **b**), 2 mm (**d**, **e**) and 800 µm (**c**)
