# Supplementary material for: Galactose induces formation of cell wall stubs and cell death in Arabidopsis roots
Source: Planta. 2022 Jul 3;256(2):26. doi: 10.1007/s00425-022-03919-x (PMC9250921; doi:10.1007/s00425-022-03919-x)
Supplement: Supplementary file 2 — Supplementary file2 (PDF 101 KB) [file 425_2022_3919_MOESM2_ESM.pdf]

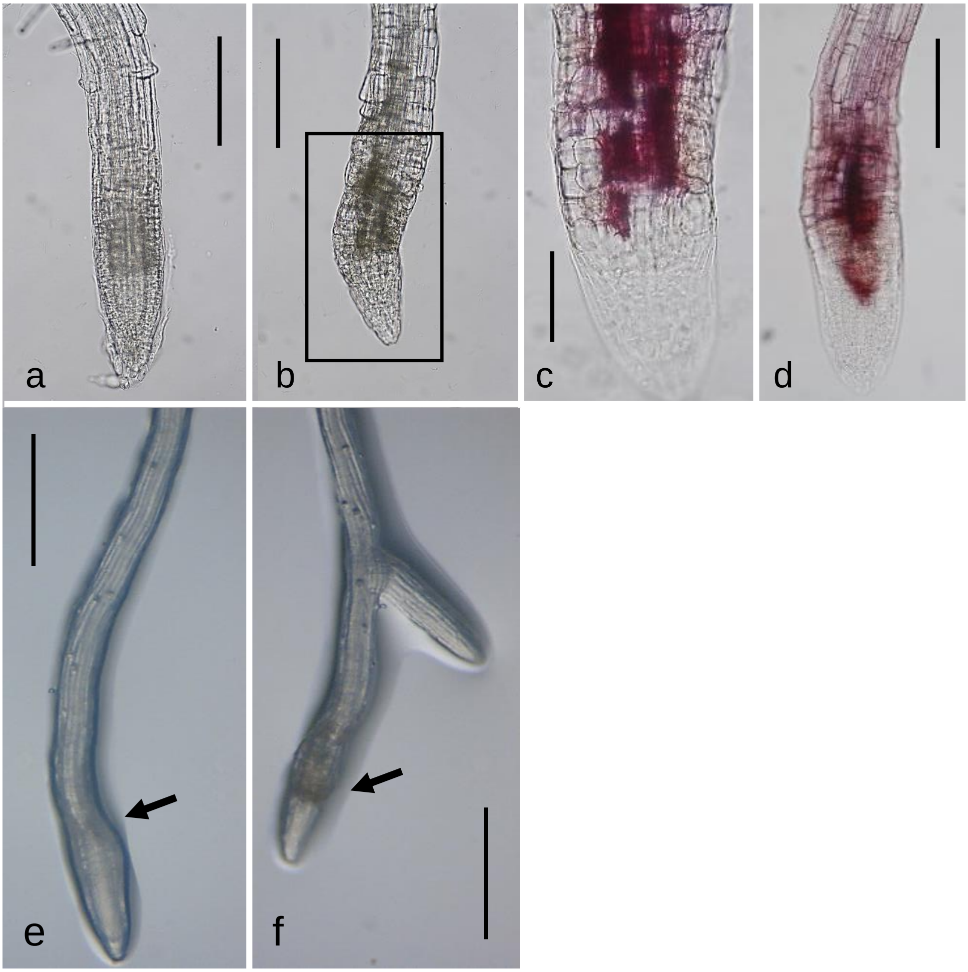

Suppl. Fig. S2 a - c Roots of seedlings grown on 0.5 mM galactose for 7 days. About 50 % of the roots look rather healthy (**a**) whereas the other 50 % show typical signs of galactose toxicity (dark and lignified regions, respectively). The phloroglucinol stained image **c** corresponds to the rectangle in **b**. **d** Phloroglucinol stained root of a seedling grown on 3 mM galactose. Note strong lignification already 3 days after sowing. **e**, **f** Roots of seedlings which were grown on 1 mM sucrose for 5 days and subsequently transferred to 1 mM galactose. Photos were taken 1 day (**e**) and 3 days (**f**) after transfer. Arrows indicate dark regions. Note newly formed lateral root in **f**. Bars 300 µm (**e**, **f**), 150 µm (**a**, **b**, **d**) and 50 µm (**c**)
