# Supplementary material for: Galactose induces formation of cell wall stubs and cell death in Arabidopsis roots
Source: Planta. 2022 Jul 3;256(2):26. doi: 10.1007/s00425-022-03919-x (PMC9250921; doi:10.1007/s00425-022-03919-x)
Supplement: Supplementary file 3 — Supplementary file3 (PDF 65 KB) [file 425_2022_3919_MOESM3_ESM.pdf]

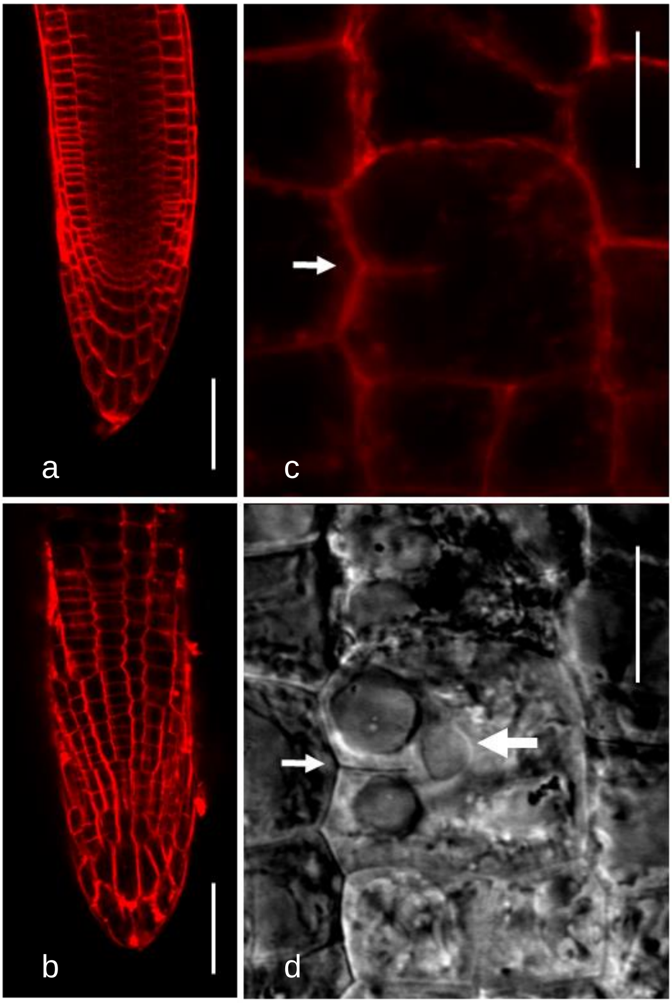

Suppl. Fig. S3 Cell pattern and cell wall stub in primary roots of *Arabidopsis thaliana* wild type grown on 1 mM sucrose for 5 days (**a**) and on 1 mM galactose for 5 (**b**) and 4 days (**c**-**d**), respectively. Note disordered cell files (**b**) and cell wall stub (thin arrows in **c** and **d**) in the roots grown on galactose. A nucleus is marked with a thick arrow in **d**. **a**-**c** show the fluorescence of FM4-64, **d** is the bright field image corresponding to **c**. Bars 50 µm (**a**, **b**) and 10 µm (**c**, **d**)
