# Supplementary material for: Galactose induces formation of cell wall stubs and cell death in Arabidopsis roots
Source: Planta. 2022 Jul 3;256(2):26. doi: 10.1007/s00425-022-03919-x (PMC9250921; doi:10.1007/s00425-022-03919-x)
Supplement: Supplementary file 4 — Supplementary file4 (PDF 136 KB) [file 425_2022_3919_MOESM4_ESM.pdf]

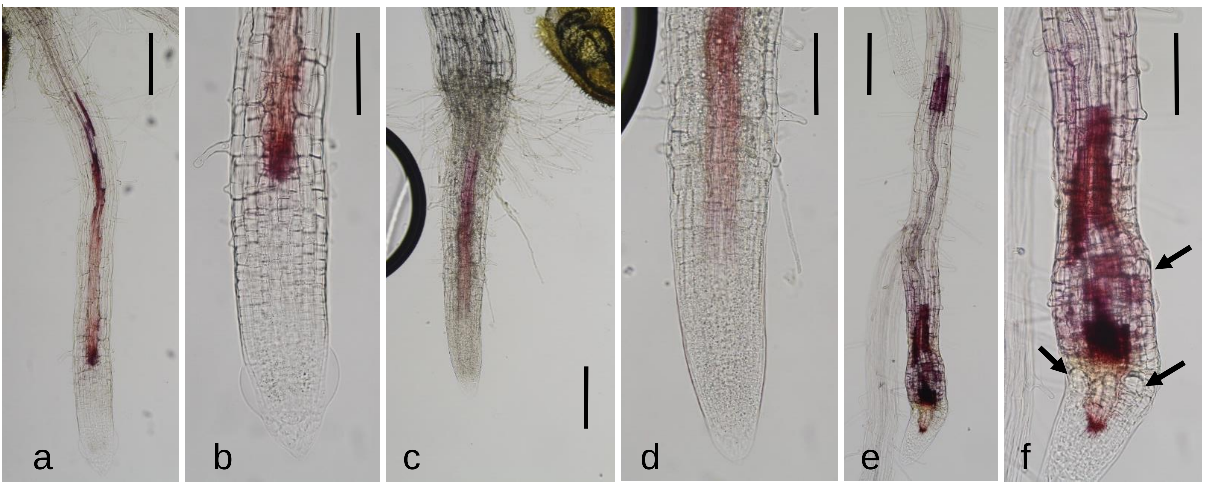

Suppl. Fig. S4 Comparison between mannose and galactose toxicity. Arabidopsis roots were stained with phloroglucinol to reveal lignification (pink). **a**, **b** Root of seedling grown on 0.3 mM mannose for 9 days. **c**, **d** Root of seedling grown on 1 mM mannose for 9 days. **e**, **f** Root grown on 1 mM galactose for 8 days. Roots grown on mannose are shorter and straighter than roots grown on galactose. They neither produce enlarged rhizodermal or cortical cells (indicated by arrows in **f**) nor cell wall stubs and lignification is restricted to the central cylinder and the endodermis. Bars 200 µm (**a**, **c**, **e**) and 100 µm (**b**, **d**, **f**)
