# Supplementary material for: Galactose induces formation of cell wall stubs and cell death in Arabidopsis roots
Source: Planta. 2022 Jul 3;256(2):26. doi: 10.1007/s00425-022-03919-x (PMC9250921; doi:10.1007/s00425-022-03919-x)
Supplement: Supplementary file 5 — Supplementary file5 (PDF 452 KB) [file 425_2022_3919_MOESM5_ESM.pdf]

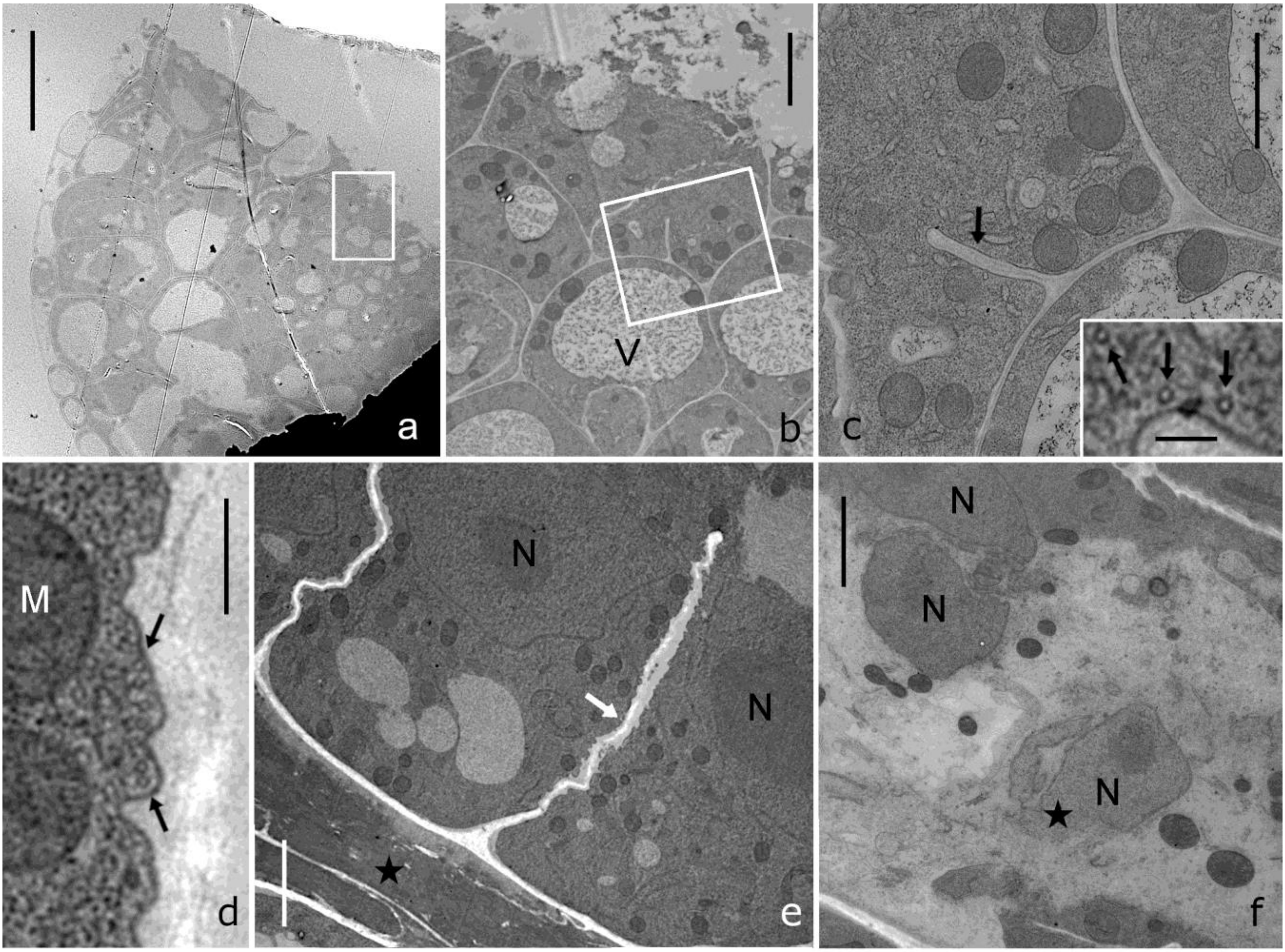

Suppl. Fig. S5 Electron microscopical images of *Arabidopsis thaliana* wild type primary roots grown on 1 mM galactose for 7 days. **a** is an overview of part of a cross section through the root tip. The rectangle indicates details shown in **b** and **c**. **b** A cell wall stub is visible in the central cylinder. **c** Higher magnification showing healthy looking cytoplasm and microtubules near the tip of the cell wall stub (inset). **d** Group of cross-sectioned microtubules (between arrows) along a cell wall stub. **e, f** Irregularly shaped nuclei in healthy looking (**e**) and in degenerated cytoplasm (**f**). Note cell wall stub (arrow in **e**), squeezed degenerated cells (asterisk in **e**) and disintegrated nuclear membrane (asterisk in **f**). Mitochondrium (M), nucleus (N), vacuole (V). Bars 20 µm (**a**), 4 µm (**b**), 2 µm (**e**, **f**), 1 µm (**c**), 250 nm (**d**) and 100 nm (inset in **c**)
