# Supplementary material for: Galactose induces formation of cell wall stubs and cell death in Arabidopsis roots
Source: Planta. 2022 Jul 3;256(2):26. doi: 10.1007/s00425-022-03919-x (PMC9250921; doi:10.1007/s00425-022-03919-x)
Supplement: Supplementary file 6 — Supplementary file6 (PDF 280 KB) [file 425_2022_3919_MOESM6_ESM.pdf]

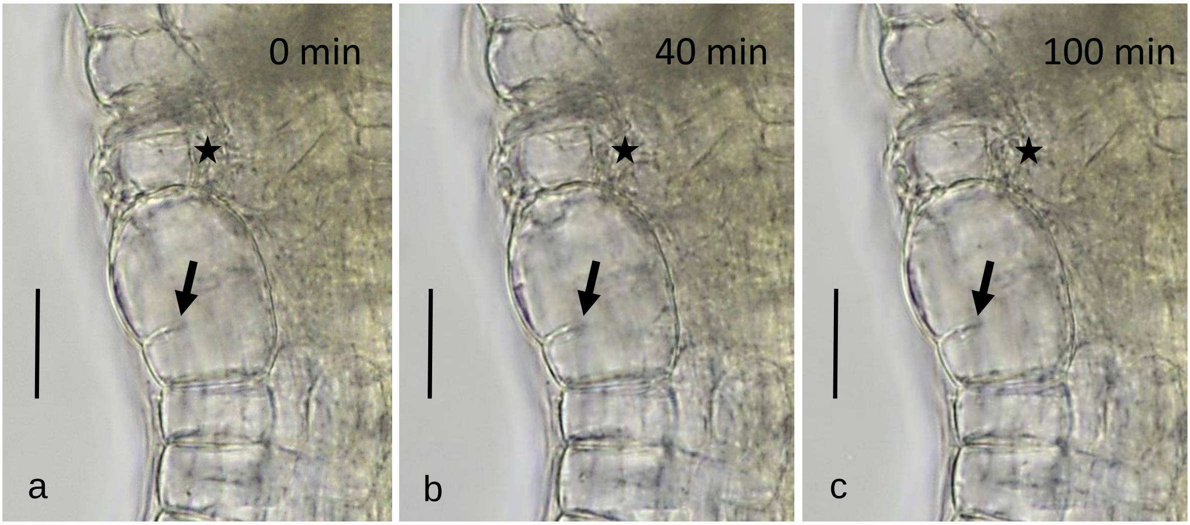

Suppl. Fig. S6 Cell wall stub (arrow) in an enlarged cell of a lateral root grown on 1mM galactose for 15 days and observed over a period of 100 minutes. The cytoplasm of the stub containing cell was actively streaming over the whole time period in contrast to that of the dead or dying neighbour cell (asterisk). Bars 10 µm
