# Supplementary material for: Galactose induces formation of cell wall stubs and cell death in Arabidopsis roots
Source: Planta. 2022 Jul 3;256(2):26. doi: 10.1007/s00425-022-03919-x (PMC9250921; doi:10.1007/s00425-022-03919-x)
Supplement: Supplementary file 7 — Supplementary file7 (PDF 270 KB) [file 425_2022_3919_MOESM7_ESM.pdf]

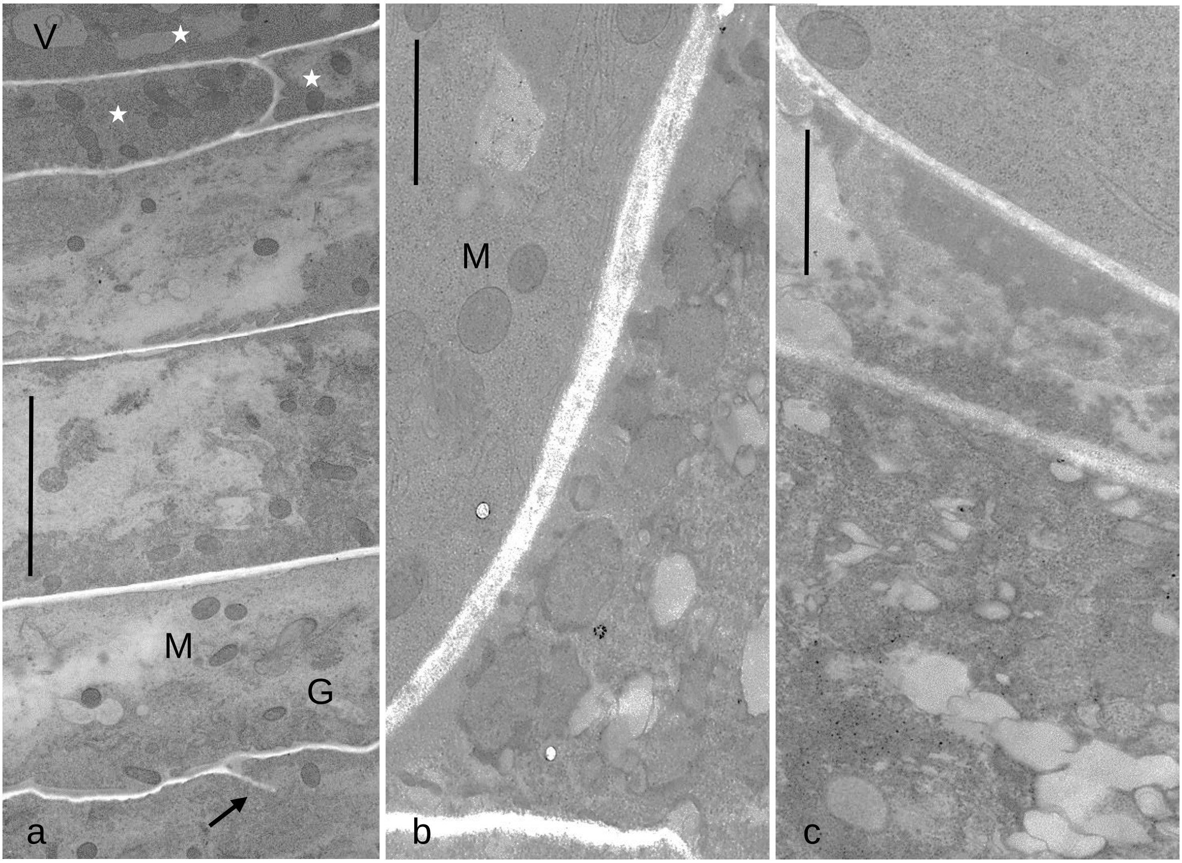

Suppl. Fig. S7 Galactose induced cell degeneration. Seedlings were grown on 1 mM galactose for 7 days before roots were fixed for electron microscopy. **a** File of cells with healthy looking cytoplasm (asterisks) and with dissolved tonoplast. A cell wall stub is seen in the lower cell (arrow). **b** Healthy looking cell with distinct organelles (left) next to a cell with putative remnants of organelles (right). **c** Healthy looking cell (above), cells with few (below) and cell without identifyable organelles (middle). Mitochondrium (M), Golgi body (G), vacuole (V). Bars 4 µm (**a**) and 1 µm (**b**, **c**)
