# Supplementary material for: Galactose induces formation of cell wall stubs and cell death in Arabidopsis roots
Source: Planta. 2022 Jul 3;256(2):26. doi: 10.1007/s00425-022-03919-x (PMC9250921; doi:10.1007/s00425-022-03919-x)
Supplement: Supplementary file 8 — Supplementary file8 (PDF 210 KB) [file 425_2022_3919_MOESM8_ESM.pdf]

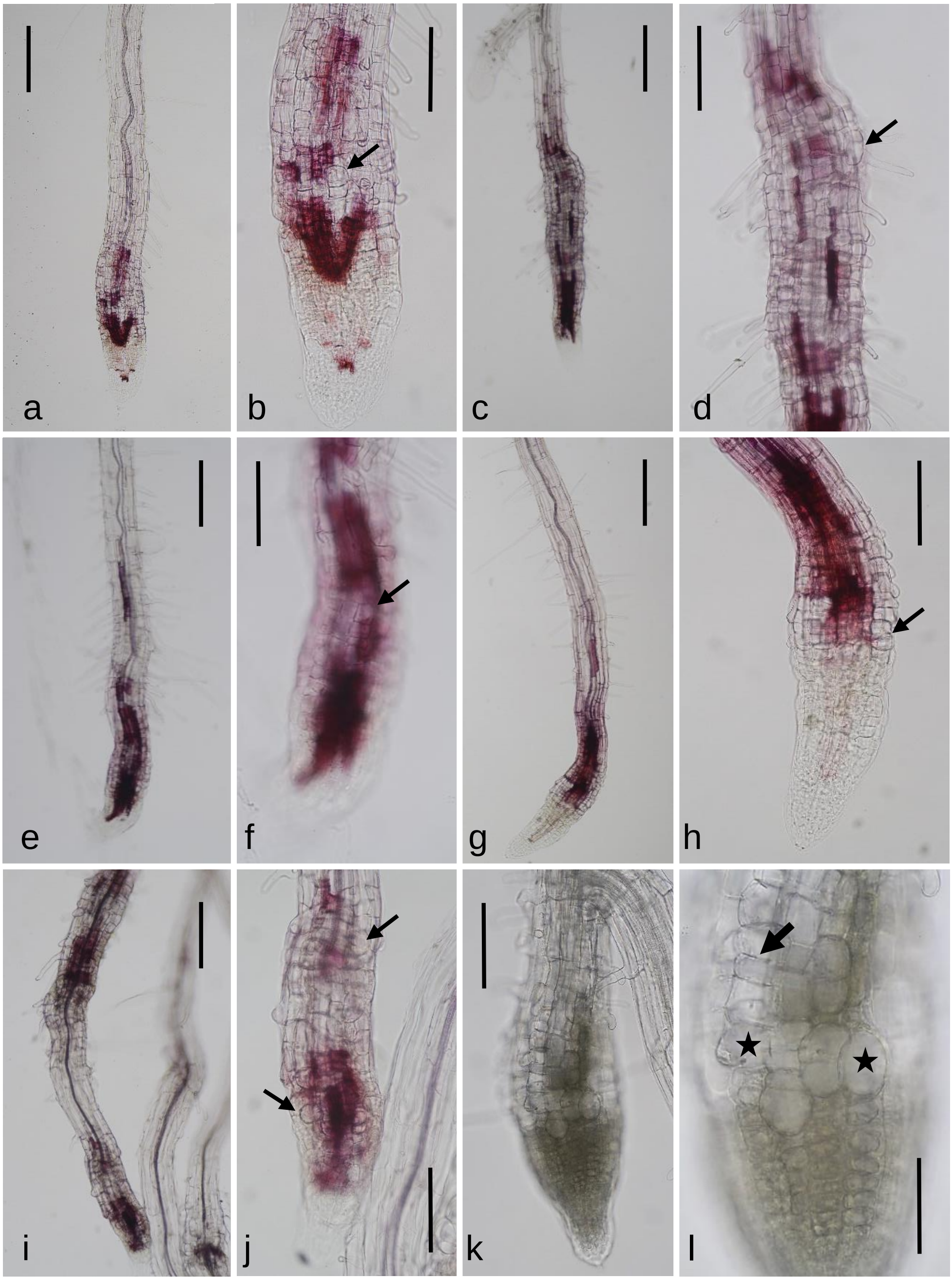

Suppl. Fig. S8 Effect of 1 mM galactose on roots grown on different media. **a**, **b** Knop/agar for 6 days. **c**, **d** MS/gelrite for 8 days. **e**, **f** MS/phytagel for 8 days. **g**, **h** MS/agar with 20x boric acid for 7 days. **i - l** MS/agar supplemented with 200 mM mannitol for 14 (**i**, **j**) and 8 days (**k**, **l**). Asterisks and thin arrows indicate enlarged cells. The thick arrow in **l** points to a cell wall stub. Bars 200 µm (**a**, **c**, **e**, **g**, **i**), 100 µm (**b**, **d**, **f**, **h**, **j**, **k**) and 50 µm (**l**)
