# Supplementary material for: Galactose induces formation of cell wall stubs and cell death in Arabidopsis roots
Source: Planta. 2022 Jul 3;256(2):26. doi: 10.1007/s00425-022-03919-x (PMC9250921; doi:10.1007/s00425-022-03919-x)
Supplement: Supplementary file 9 — Supplementary file9 (DOCX 16 KB) [file 425_2022_3919_MOESM9_ESM.docx]

**Supplementary Information**

**Suppl. Fig. S1** Seedlings of *Arabidopsis thaliana* grown on 1 mM sucrose (**a**, **d**) and on 1 mM galactose (**b**, **c**, **e**) for 11 (**a**, **b**, **d**, **e**) and 21 days (**c**), respectively. Note that the morphology of shoots is not affected by galactose. The root illustrated in **c** was stained for lignin by phloroglucinol. The root tip of the primary root (arrow) and lateral roots up to the fourth order are visible. Note that the root of an 11 day old seedling grown on galactose (**b**) is much shorter and extensively branched in comparison to the root grown on sucrose (**a**). Bars 4 mm (**a**, **b**), 2 mm (**d**, **e**) and 800 µm (**c**)

**Suppl. Fig. S2 a-c** Roots of seedlings grown on 0.5 mM galactose for 7 days. About 50 % of the roots look healthy (**a**) whereas the other 50 % show typical signs of galactose toxicity (dark and lignified regions, respectively in **b** and **c**). The phloroglucinol stained image **c** corresponds to the rectangle in **b**. **d** Phloroglucinol stained root of a seedling grown on 3 mM galactose. Note the strong lignification already 3 days after sowing. **e**, **f** Roots of seedlings grown on 1 mM sucrose for 5 days and subsequently transferred to 1 mM galactose. Photos were taken 1 day (**e**) and 3 days (**f**) after transfer. Arrows indicate dark regions. Note the newly formed lateral root in **f**. Bars 300 µm (**e, f**), 150 µm (**a, b, d**) and 50 µm (**c**)

**Suppl. Fig. S3** Cell pattern and cell wall stub in primary roots of *Arabidopsis thaliana* wild type grown on 1 mM sucrose for 5 days (**a**) and on 1 mM galactose for 5 (**b**) and 4 days (**c-d**), respectively. Note disordered cell files (**b**) and cell wall stub (thin arrows in **c** and **d**) in the roots grown on galactose. A nucleus is marked with a thick arrow in **d**. **a-c** show the red fluorescence of FM4-64, **d** is the bright field image corresponding to **c**. Bars 50 µm (**a, b**) and 10 µm (**c, d**)

**Suppl. Fig. S4** Comparison between mannose and galactose toxicity. Arabidopsis roots were stained with phloroglucinol to reveal lignification (pink). **a**, **b** Root of seedling grown on 0.3 mM mannose for 9 days. **c,** **d** Root of seedling grown on 1 mM mannose for 9 days. **e**, **f** Root grown on 1 mM galactose for 8 days. Roots grown on mannose are shorter and straighter than roots grown on galactose. They produce neither enlarged rhizodermal or cortical cells (indicated by arrows in **f**) nor cell wall stubs and lignification is restricted to the central cylinder and the endodermis. Bars 200 µm (**a, c, e**) and 100 µm (**b, d, f**)

**Suppl. Fig. S5** Electron microscopical images of *Arabidopsis thaliana* wild type primary roots grown on 1 mM galactose for 7 days. **a** is an overview of part of a cross section through the root tip. The rectangle indicates details shown in **b** and **c**. **b** A cell wall stub is visible in the central cylinder. **c** Higher magnification showing healthy looking cytoplasm and microtubules near the tip of the cell wall stub (inset). **d** Group of cross-sectioned microtubules (between arrows) along a cell wall stub. **e, f** Irregularly shaped nuclei in healthy looking (**e**) and in degenerated cytoplasm (**f**). Note the cell wall stub (arrow in **e**), squeezed degenerated cells (asterisk in **e**) and disintegrated nuclear membrane (asterisk in **f**). Mitochondrium (M), nucleus (N), vacuole (V). Bars 20 µm (**a**), 4 µm (**b**), 2 µm (**e**, **f**), 1 µm (**c**), 250 nm (**d**) and 100 nm (inset in **c**)

**Suppl. Fig. S6** Cell wall stub (arrow) in an enlarged cell of a lateral root grown on 1 mM galactose for 15 days and observed over a period of 100 minutes. The cytoplasm of the stub containing cell was actively streaming over the whole time period in contrast to that of the dead or dying neighbour cell (asterisk). Bars 10 µm

**Suppl. Fig. S7** Galactose induced cell degeneration. Seedlings were grown on 1 mM galactose for 7 days before roots were fixed for electron microscopy. **a** File of cells with healthy looking cytoplasm (asterisks) and with dissolved tonoplast. A cell wall stub is seen in the lower cell (arrow). **b** Healthy looking cell with distinct organelles (left) next to a cell with putative remnants of organelles (right). **c** Healthy looking cell (above), cells with few (below) and cell without identifyable organelles (middle). Mitochondrium (M), Golgi body (G), vacuole (V). Bars 4 µm (**a**) and 1 µm (**b, c**)

**Suppl. Fig. S8** Effect of 1 mM galactose on roots grown on different media. **a**, **b** Knop/agar for 6 days. **c**, **d** MS/gelrite for 8 days. **e**, **f** MS/phytagel for 8 days. **g**, **h** MS/agar with 20x boric acid for 7 days. **i - l** MS/agar supplemented with 200 mM mannitol for 14 (**i, j**) and 8 days (**k, l**), respectively. Asterisks and thin arrows indicate enlarged cells. The thick arrow in **l** points to a cell wall stub. Roots shown in **a-j** were stained with phloroglucinol to reveal lignification (pink). Bars 200 µm (**a, c, e, g, i**), 100 µm (**b, d, f, h, j, k**) and 50 µm (**l**)
